# Human ASPL/TUG interacts with p97 and complements the proteasome mislocalization of a yeast *ubx4* mutant, but not the ER-associated degradation defect

**DOI:** 10.1186/1471-2121-15-31

**Published:** 2014-07-31

**Authors:** Louise Madsen, Karen Molbæk, Ida B Larsen, Sofie V Nielsen, Esben G Poulsen, Peter S Walmod, Kay Hofmann, Michael Seeger, Chen-Ying Chien, Rey-Huei Chen, Franziska Kriegenburg, Rasmus Hartmann-Petersen

**Affiliations:** 1 Department of Biology, University of Copenhagen, Ole Maaløes Vej 5, Copenhagen N DK-2200, Denmark; 2 Department of Neuroscience and Pharmacology, University of Copenhagen, Symbion, Box 39, Fruebjergvej 3, Copenhagen Ø DK-2100, Denmark; 3 Institute for Genetics, University of Cologne, Cologne D-50674, Germany; 4 Institute for Biochemistry, Charité Universitätsmedizin, Berlin D-10117, Germany; 5 Institute of Molecular Biology, Academia Sinica, Taipei 11529, Taiwan

**Keywords:** Ubiquitin, Proteasome, Chaperone, Degradation

## Abstract

**Background:**

In mammalian cells, ASPL is involved in insulin-stimulated redistribution of the glucose transporter GLUT4 and assembly of the Golgi apparatus. Its putative yeast orthologue, Ubx4, is important for proteasome localization, endoplasmic reticulum-associated protein degradation (ERAD), and UV-induced degradation of RNA polymerase.

**Results:**

Here, we show that ASPL is a cofactor of the hexameric ATPase complex, known as p97 or VCP in mammals and Cdc48 in yeast. In addition, ASPL interacts *in vitro* with NSF, another hexameric ATPase complex. ASPL localizes to the ER membrane. The central area in ASPL, containing both a SHP box and a UBX domain, is required for binding to the p97 N-domain. Knock-down of ASPL does not impair degradation of misfolded secretory proteins via the ERAD pathway. Deletion of *UBX4* in yeast causes cycloheximide sensitivity, while *ubx4 cdc48-3* double mutations cause proteasome mislocalization. ASPL alleviates these defects, but not the impaired ERAD.

**Conclusions:**

In conclusion, ASPL and Ubx4 are homologous proteins with only partially overlapping functions. Both interact with p97/Cdc48, but while Ubx4 is important for ERAD, ASPL appears not to share this function.

## Background

The protein, named alveolar soft part sarcoma locus (ASPL), was first described as part of an oncogenic fusion protein with a transcription factor in alveolar soft part sarcoma tumors [1]. The normal, unfused ASPL protein contains an N-terminal UBL domain and a C-terminal UBX domain and orthologues are present in most eukaryotic species. However, the function of the ASPL protein remained unknown until it appeared in a functional screen for proteins involved in insulin-stimulated redistribution of the glucose transporter GLUT4 [2]. It was shown that ASPL binds GLUT4 directly and sequesters GLUT4 to intracellular vesicles in the absence of insulin, and ASPL was thus also named TUG for “tether containing UBX domain for GLUT4” [2]. Further studies revealed that depletion of ASPL/TUG by siRNA also enhances GLUT4 translocation to the plasma membrane and glucose uptake in adipocytes [3]. More recently, ASPL/TUG was shown to regulate assembly of the Golgi compartment [4].

Previous studies have linked both integral UBL domain proteins [5] and UBX domain proteins [6] to the ubiquitin-proteasome system, and since ASPL contains both, it is likely also to play a role in intracellular proteolysis. The structure of the ASPL/TUG UBL domain has been solved [7]. The structure shows the typical ubiquitin-like β-grasp fold, although some differences to ubiquitin, in particular the lack of the hydrophobic patch surrounding Ile44, were noted. Some UBL domain-containing proteins, such as human BAG-1 [8] and yeast Rad23 [9], utilize their UBL domains to interact with subunits of the 26S proteasome. However, the UBL domain is not a general proteasome-interacting domain [10] and binding to unrelated components has been reported [11]. In the case of ASPL, the UBL domain was recently proposed to become cleaved and function as a novel ubiquitin-like modifier [12].

The UBX domains also resemble ubiquitin and ubiquitin-like domains [13], and with few exceptions, all characterized UBX domains are implicated in binding to the AAA (ATPase associated with various activities) ATPase, known as p97 or valosin-containing protein (VCP) in mammals and Cdc48 in yeast [6,14,15]. Structurally, p97 resembles another AAA type ATPase specifically involved in vesicle fusion, named NSF [16]. The p97 ATPase is a ring-shaped homohexameric chaperone-like complex [17-21]. The monomer is a phylogenetically highly conserved protein that contains two AAA modules, called D1 and D2, that couple coordinated ATP-hydrolysis to conformational changes of the hexameric complex [22]. The ATP-driven conformational changes allow p97 to physically disassemble protein complexes and segregate proteins from their binding partners [23,24]. This “segregase” activity [25,19] is probably limited to ubiquitylated proteins and is essential for a number of cellular pathways, including membrane fusion [26], protein degradation [27,28] and transcription factor maturation through limited degradation [29,25]. Although p97 may bind ubiquitylated substrates directly [25], a series of p97 cofactors that recruit and/or process substrates has been characterized [30]. Functionally, these cofactors are diverse, and each probably directs p97 to a particular cell function. For instance, the UBX domain proteins p47 and Ubxd7 direct p97 to functions in membrane fusion [26] and protein degradation [15,31], respectively.

Previous studies have also connected p97 and its cofactors with the degradation of misfolded proteins, derived from endoplasmic reticulum (ER), via the ER-associated degradation (ERAD) pathway [32,33]. In this degradation pathway, misfolded secretory proteins are first recognized by ER luminal chaperones. Then, the proteins are retrotranslocated back to the cytoplasm, ubiquitylated, and finally degraded by the 26S proteasome [34]. The energy used to extract the proteins is provided by p97-catalyzed ATP-hydrolysis.

The putative yeast orthologue of the human UBX-domain protein ASPL, Ubx4, has been shown to regulate ERAD [35], and UV-induced degradation of RNA polymerase [36]. More recently, it was shown that budding yeast *cdc48* and *ubx4* double mutants mislocalize 26S proteasomes into foci at the nuclear envelope [37]. Moreover, *ubx4* null mutants display synthetic lethality with deletion mutants in the *rpn4* transcriptional activator for proteasome subunits and *ump1*, encoding a proteasome-assembly chaperone [37], suggesting that Ubx4 regulates proteasome maturation and trafficking.

For our understanding of p97, it is important that we obtain more detailed knowledge of its various cofactors, of which at least 20 have been found so far [30]. Using a yeast two-hybrid approach, we identified ASPL as a p97 cofactor. Our characterization of ASPL revealed that ASPL is a widely expressed protein that binds directly to p97 and NSF. Although human ASPL only displays weak sequence similarity to its putative yeast orthologue, Ubx4, expression of human ASPL in a budding yeast *ubx4*Δ mutant partially rescues the cycloheximide sensitivity and proteasome mislocalization phenotypes in *ubx4*Δ strains. This suggests that ASPL is a functional orthologue of yeast Ubx4. However, the ERAD defect in the *ubx4* null mutant is not affected by ASPL expression, and knock-down of ASPL expression in human cells does not lead to a stabilization of the model ERAD substrates, CD3δ and TCRα. Collectively, this suggests that human ASPL and budding yeast Ubx4 are multifunctional proteins that share some, but not all functions.

## Results and discussion

### ASPL is a conserved eukaryotic protein that interacts with p97

**Figure 1:**
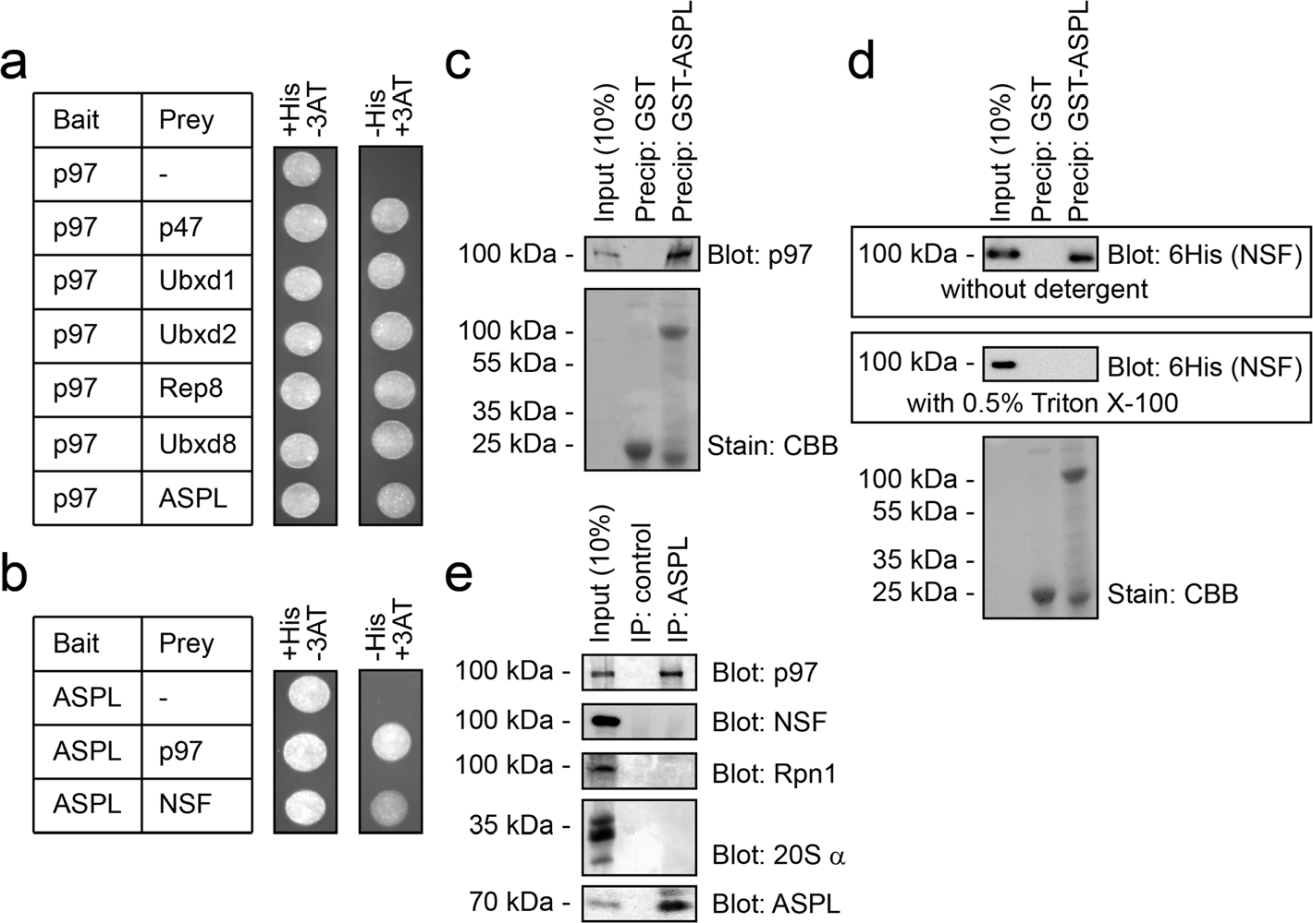
**ASPL interacts with p97 via the UBX domain. (a)** Yeast two-hybrid analyses of p97 using the *HIS3* reporter gene. Co-transformation of p97 bait with the indicated p97 binding partner preys supported cell growth under conditions selecting for interaction (in the absence of histidine and the presence of 25 mM 3-aminotriazol (3AT)) (right panel). An empty prey vector served as a negative control. **(b)** Yeast two-hybrid analyses of ASPL using the *HIS3* reporter gene. Co-transformation of ASPL bait with p97 or NSF preys supported cell growth under conditions selecting for interaction (in the absence of histidine and the presence of 25 mM 3-aminotriazol (3AT)) (right panel). An empty prey vector served as a negative control. **(c)** Purified 6His-tagged p97 was incubated with GST or GST-tagged ASPL and precipitated with glutathione (GSH) Sepharose. Bound proteins were analyzed by SDS-PAGE and blotting using antibodies to p97 (upper panel) Even loading was checked by staining with Coomassie Brilliant Blue (CBB) (lower panel). **(d)** Purified 6His-tagged NSF was incubated with GST or GST-tagged ASPL and precipitated with glutathione (GSH) Sepharose. Bound proteins were analyzed by SDS-PAGE and blotting using antibodies to the 6His-tag on NSF (upper panels). Even loading was checked by staining with Coomassie Brilliant Blue (CBB) (lower panel). Interaction to NSF was only evident when no detergents were included in the buffer system. In the presence of 0.5% Triton X-100 no interaction between ASPL and NSF was observed. **(e)** MelJuSo cell lysates were used in immunoprecipitation (IP) experiments with antibodies to ASPL and Protein A Sepharose or as a control Protein A Sepharose beads only. SDS-PAGE and blotting revealed that ASPL co-precipitated p97, but not NSF or the Rpn1 or α subunits of the 26S proteasome.

In a yeast two-hybrid screen of a HeLa cell cDNA library, using human p97 as a bait, several UBX domain proteins were found. These included p47, ASPL as well as Ubxd1, Ubxd2, Ubxd8, and Rep8 (Figure 1a). Previously, most of these proteins have been described in the context of p97-binding, and connected with p97-relevant functions [26,15,38,39]. The human ASPL protein is 77% identical with its murine orthologue and 47% identical with its orthologue in *Xenopus laevis* (Additional file 1: Figure S1). ASPL is found in most eukaryotes. The closest homologue to ASPL in plants is PUX1, a known p97 cofactor [40,41]. The putative budding yeast orthologue is Ubx4. However, Ubx4 is only weakly homologous to human ASPL (18% overall sequence identity) (Additional file 1: Figure S2). ASPL contains a UBL domain near the N-terminus, and a UBX domain near the C-terminus (Additional file 1: Figure S1). In addition, by sequence analyses we found a low homology UBX domain at position 89–169 which we named LHU for low homology UBX domain and a putative p97-binding SHP-box between residues 244 and 254. Curiously, the SHP box is not conserved in budding yeast Ubx4 (Additional file 1: Figure S2). The UBX domain and SHP box are regarded as general p97-interacting modules, indicating that ASPL was a valid target of p97 in the yeast two-hybrid screen.

To obtain further information on ASPL interacting proteins, we performed another round of yeast two-hybrid screening, now using full length ASPL as bait. Here the isolated clones encoded either p97 or the N-ethylmaleimide (NEM) sensitive factor (NSF). When comparing the p97 and NSF clones, we noticed that p97 activated the *HIS3* reporter gene stronger than did NSF (Figure 1b).

In order to confirm the yeast two-hybrid interactions, GST and GST-tagged ASPL (Figure 1c and Figure 1d) were expressed and purified from *E. coli*. The fusion proteins were used in precipitation experiments with 6His-tagged p97 and 6His-tagged NSF, produced in *E. coli*. GST-ASPL precipitated p97 (Figure 1c) and NSF (Figure 1d) whereas, under the same conditions, GST did not, thus confirming that ASPL interacts directly with p97 and NSF. However, we noted that the ASPL-NSF interaction was lost when detergents were included in the buffer system (Figure 1d).

To determine if the identified protein-protein interactions also occur *in vivo,* between the endogenous proteins, we immunoprecipitated ASPL from mammalian cell extracts and analyzed the precipitated material by blotting for p97 and NSF. Indeed, the interaction between p97 and ASPL was also evident between the endogenous proteins (Figure 1e). However, we did not detect any NSF in the immunoprecipitates (Figure 1e), even after chemical cross-linking (not shown). This suggests that either the ASPL-NSF interaction *in vivo* is weak and/or transient, or that the proteins do not interact *in vivo*. We therefore decided not pursue the interaction between NSF and ASPL further. Since ASPL contains a UBL domain, and some UBL domain proteins function as proteasome co-factors [10], we also tested if ASPL interacts with 26S proteasomes. However, since neither the Rpn1 subunit of the 19S proteasome complex nor the α-subunits 20S proteasome complex were recovered in the ASPL immunoprecipitate, we conclude that APSL is not associated with 26S proteasomes in mammalian cells (Figure 1e).

**Figure 2:**
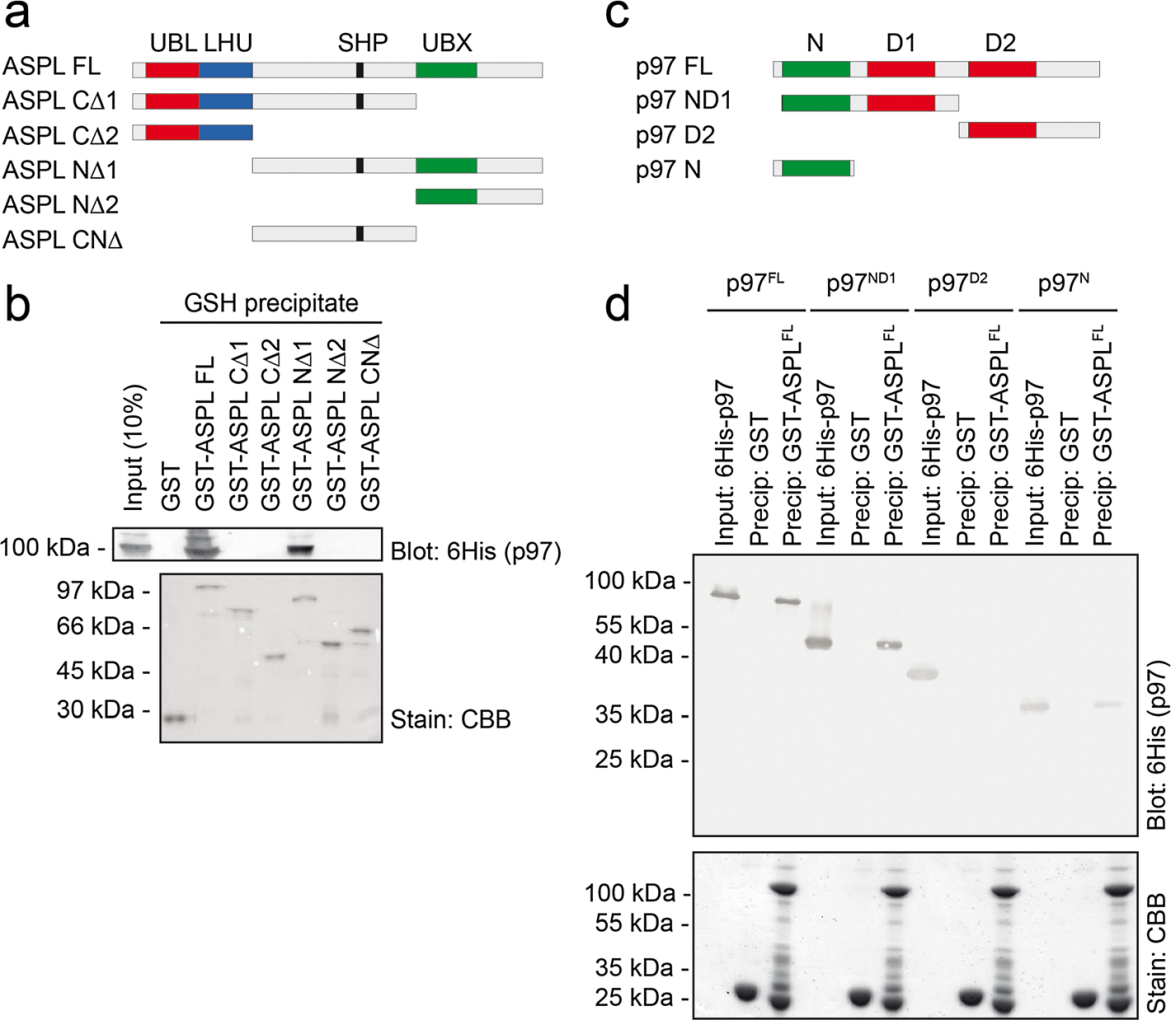
**ASPL interacts with the p97 N-domain. (a)** Schematic diagram of the ASPL domain organization and the various truncations used in the precipitation experiments. **(b)** Purified 6His-tagged p97 was incubated with GST or the indicated GST-tagged ASPL truncations and precipitated with glutathione (GSH) Sepharose. Bound proteins were analyzed by SDS-PAGE and blotting using antibodies to the 6His-tag on p97 (upper panel). Even loading was checked by staining with Coomassie Brilliant Blue (CBB) (lower panel). **(c)** Schematic diagram of the p97 domain organization and the various truncations used in the precipitation experiments. **(d)** Purified 6His-tagged p97 and p97 truncations were incubated with GST and GST-tagged ASPL before precipitation and analysis by SDS-PAGE and blotting using antibodies specific for the 6His-tagged p97 proteins (upper panel). Even loading was checked by staining with Coomassie Brilliant Blue (CBB) (lower panel).

Next, we decided to map the interaction between ASPL and p97. Further GST-precipitation experiments using full length ASPL and various ASPL truncations (Figure 2a) revealed that the C-terminal UBX domain was not able to precipitate p97 (Figure 2b). Curiously, the construct, lacking the UBX domain, did also not co-precipitate p97 (Figure 2b), indicating that efficient binding to ASPL requires an extended central area containing multiple interaction areas. Further truncations revealed that the N-terminal half of ASPL, containing the UBL and LHU domains, failed to interact with p97 (Figure 2b), while the central area containing the SHP box and UBX domain did bind p97 (Figure 2b), although with a reduced affinity. Further co-precipitation experiments using truncated 6His-tagged versions p97 (Figure 2c) and full length GST-tagged ASPL revealed that ASPL interacts with the p97 N-domain (Figure 2d). We conclude that ASPL associates with the p97 N-domain via the central area, containing the SHP box and UBX domain.

### ASPL is a widely expressed protein and localizes to the ER-membrane

**Figure 3:**
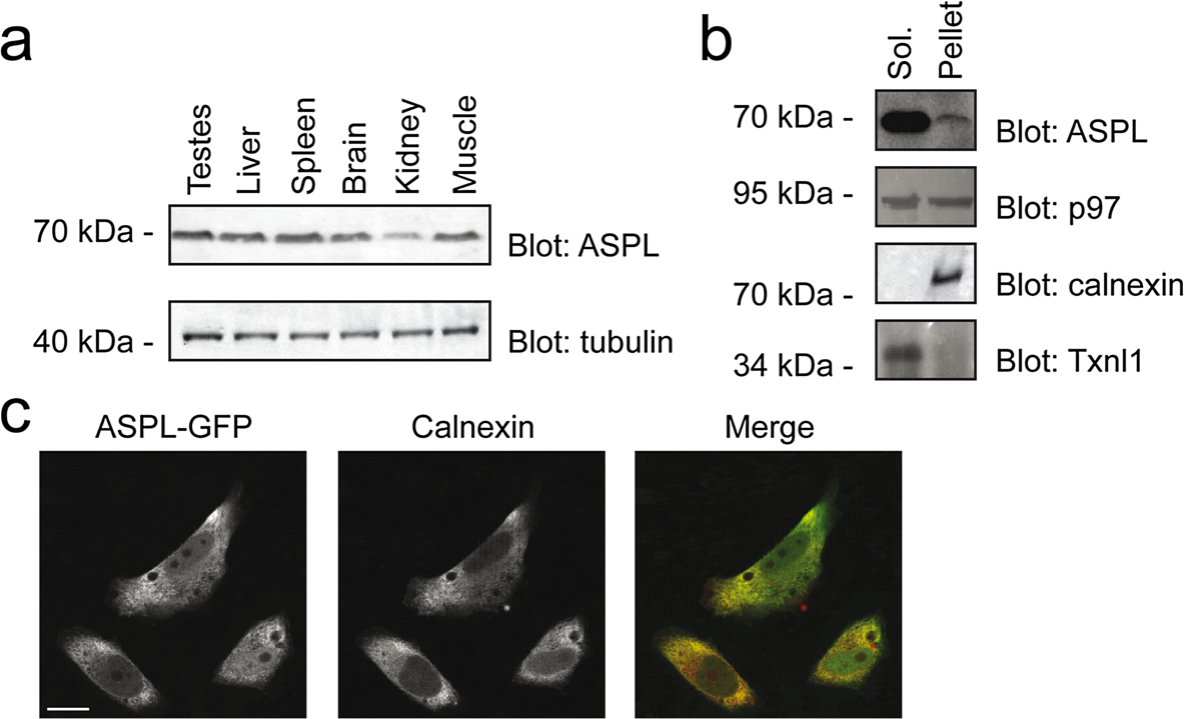
**Expression and localization of ASPL. (a)** The rat tissues were analyzed by SDS-PAGE and blotting using antibodies specific for ASPL (upper panel) and tubulin (lower panel). ASPL was present in most tissues, but less abundant in kidney. **(b)** HeLa cells lysates were separated by centrifugation into a soluble (Sol.) and insoluble (Pellet) fraction and analyzed by SDS-PAGE and blotting using antibodies to the listed proteins. Calnexin served as a control for an insoluble (transmembrane) protein, while Txnl1 served as a control for a soluble protein. **(c)** Confocal micrographs of HeLa cells transfected to express ASPL with a C-terminal GFP-tag. The localization of ASPL in formaldehyde-fixed cells was detected with anti-GFP antibodies (green) (left panel), and compared with the localization of the ER protein calnexin (red) (middle panel). In the merged image (right panel), the signals overlap partially (yellow). The presented cells are derived from different confocal scans. Size-bar = 10 μm.

Since p97 and NSF are connected with a range of basic cellular functions, their cofactors are expected to be widely expressed. However, some p97 cofactors display a very narrow tissue expression profile [39]. To determine the tissue distribution of ASPL on the protein level, we separated protein extracts from various rat tissues by SDS-PAGE and probed blots for the presence of ASPL. We found that ASPL was expressed in all the tested tissues, but was less abundant in kidney (Figure 3a).

Next, we sought to determine the subcellular localization of ASPL. Separation of cell lysates into a soluble and insoluble fraction revealed that ASPL is largely soluble (Figure 3b).

Recently, endogenous ASPL was shown to co-localize with ERGIC-53 at the ER-Golgi intermediate compartment [4]. In our hands, the commercially available antibodies to ASPL were not functional for immunofluorescence microscopy. HeLa cells were therefore transiently transfected to express full length ASPL with a C-terminal GFP-tag. The GFP signal appeared throughout the cells, but was increased at the ER, as determined by concurrent staining of the ER protein, calnexin (Figure 3c).

### Effect of knock-down of ASPL expression

**Figure 4:**
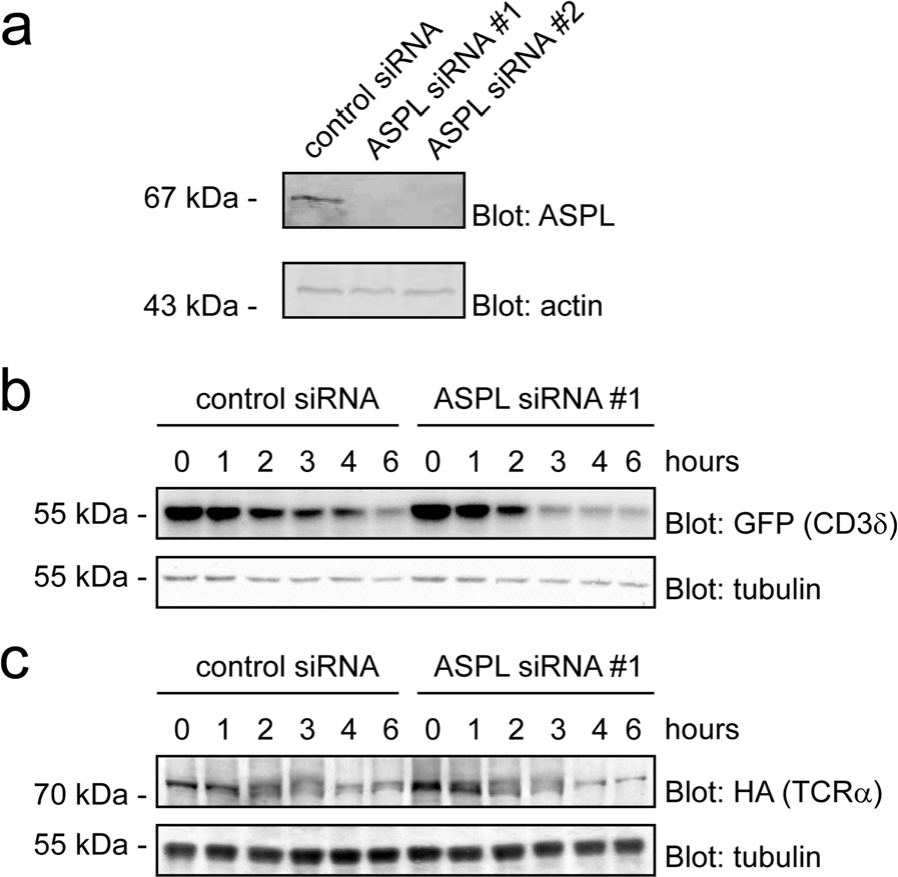
**ASPL does not influence the degradation of model ERAD substrates. (a)** MelJuSo cells were transfected with control siRNA or two different siRNAs, specific for ASPL. After 3 days, the expression of the indicated proteins was analyzed by SDS-PAGE and blotting. Actin served as a loading control. **(b)** MelJuSo cells expressing YFP-tagged CD3δ were analyzed in cultures treated with cycloheximide. At the indicated times the amount of CD3δ-YFP was analyzed by SDS-PAGE and Western blotting using the indicated antibodies. Antibodies to α-tubulin were used for the loading control. **(c)** HeLa cells expressing HA-tagged TCRα were analyzed in cultures treated with cycloheximide. At the indicated times the amount of TCRα-HA was analyzed by SDS-PAGE and Western blotting using the indicated antibodies. Antibodies to α-tubulin were used for the loading control.

Since ASPL is a putative orthologue of yeast Ubx4, which has been connected to ERAD [35], the ERAD pathway might also be impaired in cells with a decreased amount of ASPL. To test this prediction, ASPL expression was knocked-down with siRNA (Figure 4a), and the degradation kinetics of the model ERAD substrates CD3δ-YFP (Figure 4b) and TCRα-HA (Figure 4c) were analyzed. We observed no significant differences in these substrates degradation kinetics when ASPL was lacking (Figure 4b and Figure 4c). We therefore conclude that ASPL, unlike its putative yeast orthologue Ubx4, is not involved in ERAD, at least with these substrates.

### ASPL complements some defects of a budding yeast ubx4Δ strain

**Figure 5:**
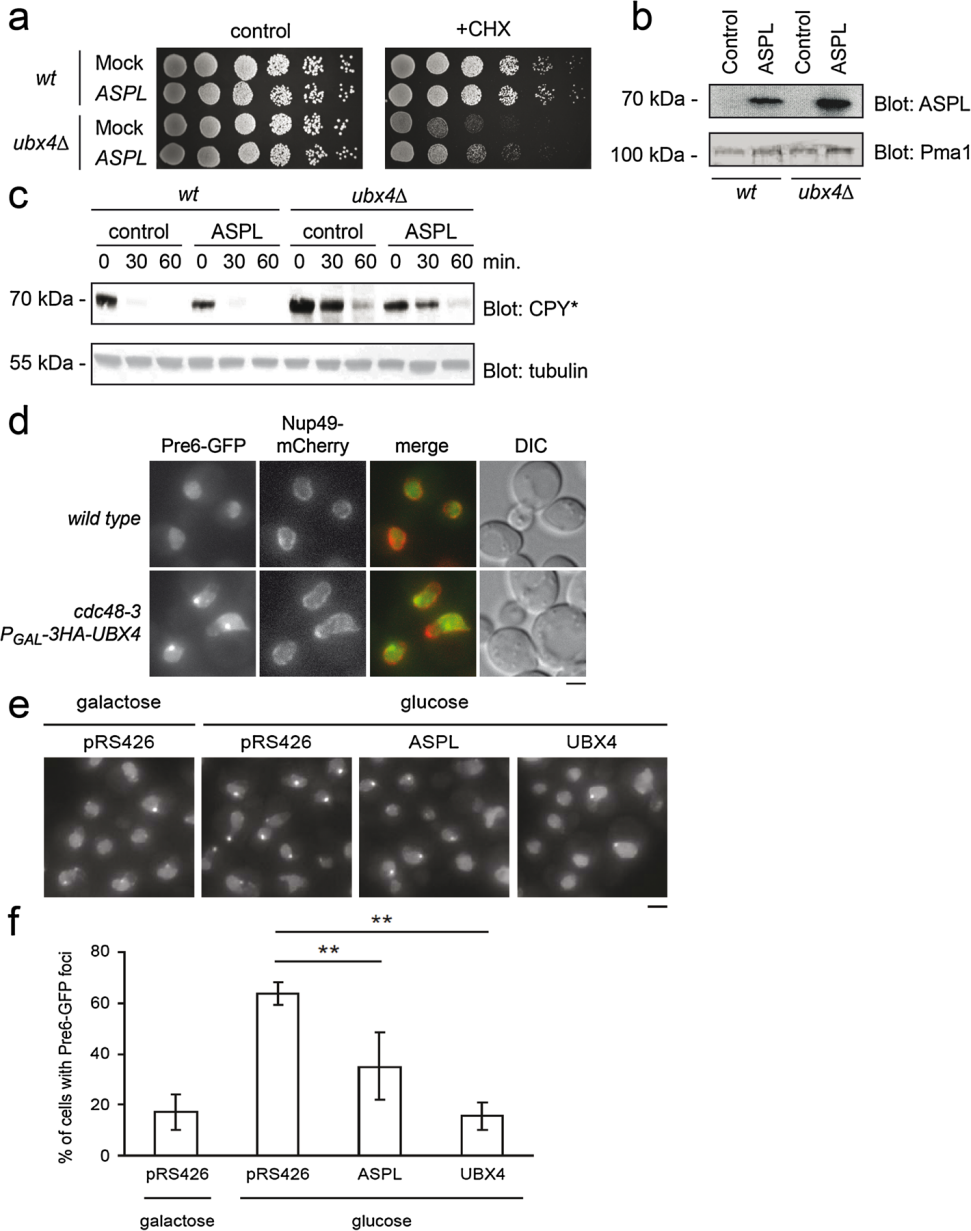
**Human ASPL partially suppresses some defects of a yeast ubx4Δ mutant. (a)** Serial dilutions of wild type or *ubx4*Δ strains transformed with either a control plasmid (Mock) or an ASPL expression vector (*ASPL*) were spotted onto solid media with or without 0.2 μg/mL cycloheximide (CHX). The plates were incubated at 30°C until colonies formed. **(b)** Extracts from the indicated strains were resolved by SDS-PAGE and blotting using antibodies to ASPL and, as a loading control, the constitutive Pma1. **(c)** Wild type (wt) and *ubx4*Δ cells expressing CPY* were treated with cycloheximide. At the indicated times the amount of CPY* was analyzed by SDS-PAGE and Western blotting using the indicated antibodies. **(d)** Wild-type yeast and *cdc48-3 P*_*GAL*_*-3HA-UBX4* mutant expressing Pre6-GFP and nuclear envelope marker Nup49-mCherry were grown in synthetic medium containing glucose and analyzed by fluorescence microscopy. **(e)** Yeast 2 μ plasmids carrying *ASPL* or *UBX4* genes were transformed into *PRE6-GFP cdc48-3 P*_*GAL*_*-3HA-UBX4* strain. Transformation with the empty vector pRS426 serves as negative control. Cells were grown in synthetic medium containing galactose or glucose to induce or repress, respectively, *UBX4* and analyzed by fluorescence microscopy. **(f)** The percentage of cells containing Pre6-GFP foci were quantified and shown as average and standard deviation for three independent experiments. Bar, 2 μm. (**, p < 0.01).

Yeast mutants lacking Ubx4 have been reported to be sensitive to stress conditions, including high temperatures, reducing agents and cycloheximide [35]. To test whether ASPL is a functional orthologue of yeast Ubx4, we examined whether expression of human ASPL could rescue the cycloheximide sensitive phenotype of the *UBX4* deletion mutant in *S. cerevisiae*. To this end, wild type and *ubx4*Δ strains were transformed with an expression plasmid encoding ASPL and, as a control, an empty vector. Under normal conditions, cell growth was not affected by loss of Ubx4 or expression of ASPL (Figure 5a). However, expression of ASPL (Figure 5b) partially rescued the cycloheximide-dependent growth defect of the *ubx4*Δ mutant (Figure 5a), revealing that ASPL, at least in part, can remedy the lack of Ubx4 in *ubx4*Δ cells. To examine if ASPL expression could also alleviate the ERAD defect in the *ubx4*Δ strain, we followed the degradation of the ERAD substrate CPY*. As expected, we found that deletion of *UBX4* led to an impaired degradation of CPY* (Figure 5c). However, ectopic expression of ASPL had no effect on the CPY* degradation (Figure 5c).

More recently, Ubx4 was shown to regulate proteasome localization along with Cdc48 [37]. In mammalian cells, proteasomes are distributed throughout the cytosol and nucleus [42], while in budding yeast, proteasomes are enriched in the nucleus [43]. We found that proteasomes, as visualized by Pre6-GFP, concentrated to a discrete spot when *UBX4* expression was repressed through *GAL* promoter in the *cdc48-3* strain (Figure 5d). This mislocalization was fully suppressed by ectopic expression of *UBX4*, and partially suppressed by expression of ASPL (Figure 5,e and f), revealing that this function is conserved between the yeast and human proteins.

Finally, we tested if ASPL also affected proteasome localization in human cells. Under normal conditions we found proteasomes fairly evenly spread throughout the cytosol and nucleus (Additional file 1: Figure S3), and this localization was not changed upon knock-down of ASPL expression (Additional file 1: Figure S3). Hence, either ASPL does not regulate the subcellular localization of proteasomes in mammalian cells, or perhaps this function of ASPL is redundant with other components in higher eukaryotes.

## Conclusions

Recently, it was reported that ASPL is required for reassembly of the Golgi apparatus [4]. On most points, e.g. association with p97, interaction mapping and subcellular localization, our data largely agree with these published results. In addition, we show here that human ASPL can partially complement the cycloheximide sensitive phenotype and proteasome mislocalization of budding yeast mutants, devoid of Ubx4. However, heterologous ASPL expression does not restore ERAD in the *ubx4*Δ mutant. Thus, unlike Ubx4, human ASPL is not involved in ERAD, but rather shares some other cellular functions with Ubx4, including regulation of proteasome localization.

## Methods

### Buffers

The buffers were: Buffer A, 25 mM Tris/HCl pH 7.5, 2 mM MgCl_2_, 2 mM ATP, 50 mM NaCl, 1 mM DTT, 10% (v/v) glycerol, 0.1% (v/v) Triton X-100. Buffer B, 33 mM Hepes pH 7.3, 150 mM potassium acetate, 10% (v/v) glycerol, 1% (w/v) DeoxyBigChap (Fluka). Buffer C, 33 mM Hepes pH 7.3, 150 mM potassium acetate, 10% (v/v) glycerol, 0.2% (w/v) DeoxyBigChap (Fluka), 1 mg/mL BSA. Buffer D, 25 mM Tris/HCl pH 7.5, 2 mM ATP, 50 mM NaCl, 1 mM DTT, 10% (v/v) glycerol. In cell lysates, all buffers were supplemented with Complete protease inhibitor cocktail tablets (EDTA-free, Roche) and 1 mM PMSF.

### Plasmids and expression

For expression of recombinant ASPL, full-length cDNA and various truncations encoding human ASPL were transferred to the appropriate Gateway destination vectors (Invitrogen). The expression constructs for human p97 were kindly provided by Prof. Hemmo H. Meyer (Zürich, Switzerland). The expression construct for 6His-tagged NSF was kindly provided by Prof. Sidney W. Whiteheart (Kentucky, USA). Transformed *E. coli* BL21*(DE3), M15 or Rosetta cells, expressing tagged protein, were lysed by sonication in one volume of buffer A for p97-related experiments and in buffer D for NSF-related experiments. The extracts were cleared by centrifugation (12000 g, 30 min) and the fusion protein was purified by standard methods.

### Yeast two-hybrid

Yeast two-hybrid screening using full length human p97 was performed on a HeLa cell cDNA library (Invitrogen) using the ProQuest yeast two-hybrid system (Invitrogen) according to the protocol supplied by the manufacturer.

### Saccharomyces cerevisiae strains and techniques

The genetic backgrounds of the *S. cerevisiae* strains used in this study were W303 (*MATα ade2-locre can1-100 his3-11, 15 leu2-3, 112 trp1-1 ura3-1 prc1-1*) and W303 *ubx4::kanMX*[35]. These strains were generously provided by Prof. Dieter H. Wolf (Stuttgart, Germany). The strains were transformed using lithium acetate with expression plasmids for human ASPL or as a control pEMBLyex4.

### Cell culture

MelJuSo cells, stably transfected to express YFP-tagged CD3δ [44], were kindly supplied by Dr. Nico P. Dantuma (Stockholm, Sweden). HeLa cells, stably transfected to express HA-tagged TCRα [45], were kindly provided by Dr. Cezary Wojcik (Evansville, USA). These cells and standard HeLa cells were maintained in Dulbecco’s modified Eagle’s minimal essential medium (DMEM) supplemented with 10% newborn-calf serum (Invitrogen) at 37°C in a humidified atmosphere containing 7.5% CO_2_.

### Electrophoresis and blotting

Proteins were separated on 7 cm x 8 cm 12.5% acrylamide gels. Proteins were transferred to BA83 (Schleicher & Schuell) nitrocellulose membranes and probed with antibodies as indicated.

### Antibodies

Antibodies and their sources were: anti-human ASPL (Abcam and Cell Signaling), anti-NSF (NSF-1 monoclonal purchased from Abcam), anti-his (Qiagen), anti-GFP (Invitrogen), anti-tubulin and anti-β-actin (Abcam), anti-human Rpn1 (p112, Enzo) and anti-20S proteasome α-subunits (MCP231, Enzo). The antibodies to p97 have been described previously [46]. Several antibodies were kindly provided by the following investigators: anti-calnexin (Prof. Ari Helenius, Zürich, Switzerland), anti-Pma1 (Prof. Per A. Pedersen, Copenhagen, Denmark), anti-CPY* (Prof. Jakob R. Winther, Copenhagen, Denmark).

### Yeast extracts and growth assays

The transformed yeast strains were grown to late exponential phase and lysed directly or used for cycloheximide decay assays prior lysis. Cell lysis was performed using NaOH. Briefly, cells were harvested and resuspended in 1–2 volumes water. A 0.6 M NaOH stock solution was added to give a final concentration of 0.1 M NaOH and the cells were incubated for 5 min at room temperature. Subsequently, the cells were pelleted and resuspended in SDS sample buffer, boiled for 20 minutes and finally sonicated for 20 seconds.

For growth assays, cells in exponential phase were washed once in water and resuspended to an OD_600nm_ of 0.4 in water. Five-fold dilution series were prepared of each culture in water and 5 μL of each dilution were spotted onto solid minimal media with or without 0.2 μg/mL cycloheximide and grown at 30°C.

### Transfection of siRNA

HeLa cells expressing HA-tagged TCRα or MelJuSo cells expressing YFP-tagged CD3δ were forward transfected with 50 nM siRNA using either DharmaFECT (Dharmacon) or Lipofectamine RNAiMAX (Invitrogen), respectively. Briefly for HeLa, exponentially growing cells were washed in PBS and incubated for 24 hours with a solution containing siRNA, 0.4% Dharmafect in DMEM supplemented with 1% calf serum. The medium was then changed to DMEM with 10% serum. For MelJuSo cells, the siRNA and Lipofectamine RNAiMAX were mixed directly in OptiMEM (Invitrogen) and carefully added to exponentially growing cell. Cycloheximide decay assays were performed 72 hours after transfection.

### Co-precipitation experiments

For *in vitro* binding studies, GST and GST-tagged ASPL variants were expressed in *E. coli* and bound to Glutathione Sepharose (GE Healthcare). Equal amounts of purified, immobilized proteins were incubated with cleared *E. coli* lysate containing either recombinant 6His-tagged p97 or 6His-tagged NSF for 2 hours at 4°C under gentle agitation. Subsequently, the lysate was removed and the beads were washed 3 times in buffer A for p97 and in buffer D for NSF pull downs. The immobilized proteins were eluted in 4 x SDS sample buffer, subjected to SDS-PAGE and visualized by Western blotting and/or Coomassie Brilliant Blue staining.

For *in vivo* binding studies, HeLa cells were lysed in buffer B under gentle agitation for 30 min for p97-related experiments. For NSF-related experiments MelJuSo cells were resuspended in buffer D and lysed via sonication. Cell extracts were cleared by centrifugation at 12000 g for 20 minutes and either incubated with Protein A Sepharose alone as a control or with an antibody to ASPL (1:100, Cell Signaling) for 30 minutes at 4°C before adding Protein A Sepharose (GE Healthcare) to the antibody containing samples. After further incubation for 1.5 hours at 4°C under gentle agitation, the supernatant was carefully removed and the beads were washed 3 times in either buffer C or in buffer D for p97 or NSF co-precipitation, respectively. Bound proteins were eluted in SDS sample buffer, subjected to SDS-PAGE and visualized by Western blotting.

### Protein degradation experiments

The degradation of CD3δ-YFP, TCRα-HA and CPY* was followed in cultures treated with cycloheximide as described [47,35].

### Differential centrifugation

Differential centrifugation was performed as described [48].

### Fluorescence microscopy of HeLa cells

For fluorescence microscopy, HeLa cells were plated in 4-well Permanox LabTek chamber slides (Nunc) and transiently transfected with plasmids for expression of ASPL and ASPL truncations with a C-terminal GFP-tag, using Lipofectamine 2000 according to the manufacturer’s instructions (Invitrogen). About 48 hours after transfection, cells were fixed in formaldehyde and stained with mouse anti-GFP and rabbit anti-calnexin in combination with Alexa 488-conjugated goat anti-mouse and Alexa 543-conjugated goat anti-rabbit antibodies (Invitrogen), respectively.

Confocal micrographs were obtained with a Radiance 2000 confocal laser scanning system (BioRad) attached to an inverted Nikon Eclipse TE 200 microscope. Scans were performed with a pixel resolution of 0.062 μm using a 60x objective, Ar and HeNe lasers, a 560 DCLP dichroic mirror, a HQ 515/30 emission filter (for Alexa 488) and an E570LP emission filter (for Alexa 543).

For the immunofluorescence microscopy of proteasomes in HeLa cells, siRNAs were transfected using RNAiMAX (Invitrogen) 48 hours prior to fixation in formalin (Sigma). The cells were then washed in PBS and permabelized in 0.2% Triton X-100 in PBS for 5 minutes at room temperature before 5 mg/mL BSA (Sigma) in 20 mM glycine in PBS was applied for 30 minutes at room temperature. The antibodies used for detection were: MCP34 to proteasome subunit α4 (Enzo) and TBP1-19 to proteasome subunit Rpt5 (Enzo) diluted 1:100, and Alexa Fluor 594 goat anti-mouse IgG (Invitrogen) diluted 1:1000. Images were acquired using a fluorescence microscope (Zeiss AxioImager Z1) and CCD camera (Hamamatsu ORCA-ER).

### Fluorescence microscopy of yeast cells

For experiments on proteasome localization, cells were first grown in synthetic medium lacking uracil to maintain the 2 μ plasmid and containing galactose to express *UBX4*. At early exponential phase, the cell culture was either maintained in the same medium or shifted to glucose medium to suppress *UBX4* for another 3 hours at 25°C. The fluorescence images were acquired with a 100x 1.4 N/A objective and CoolSNAP HQ^2^ CCD camera (Photometircs) on Olympus IX71 fluorescence microscope controlled by DeltaVision system (Applied Precision). Z-stacks of 13 optical sections with 0.5 μm spacing were collected and processed by Softworx software. Projections of maximum intensity were shown.

## Supporting data

The present article contains supporting data.

## Abbreviations

ASPL: Alveolar soft part sarcoma locus; CBB: Coomassie brilliant blue; CHX: Cycloheximide; ER: Endoplasmic reticulum; ERAD: ER-associated degradation; GSH: Glutathione; IP: Immunoprecipitation; LHU: Low homology UBX; NEM: N-ethylmaleimide; NSF: NEM sensitive factor; TUG: Tether containing UBX domain of GLUT4; VCP: Valosin-containing protein; 3AT: 3-aminotriazol.

## Competing interests

The authors declare that they have no competing interests.

## Authors’ contributions

L.M., K.M., I.B.L., S.V.N., E.G.P., P.S.W., C.C. and F.K. performed the experiments. K.H. performed the bioinformatic analyses. F.K., M.S., R.C. and R.H.P. analyzed the data. F.K., L.M., R.C. and R.H.P. wrote the paper. All authors read and approved the final manuscript.

## Supplementary Material

Additional file 1 Supplementary figures and legends. Click here for file
