# Supplementary material for: Human ASPL/TUG interacts with p97 and complements the proteasome mislocalization of a yeast ubx4 mutant, but not the ER-associated degradation defect
Source: BMC Cell Biol. 2014 Jul 31;15:31. doi: 10.1186/1471-2121-15-31 (PMC4124494; doi:10.1186/1471-2121-15-31)
Supplement: Additional file 1 — Supplementary figures and legends. [file 1471-2121-15-31-S1.pdf]

Supplementary figures and legends

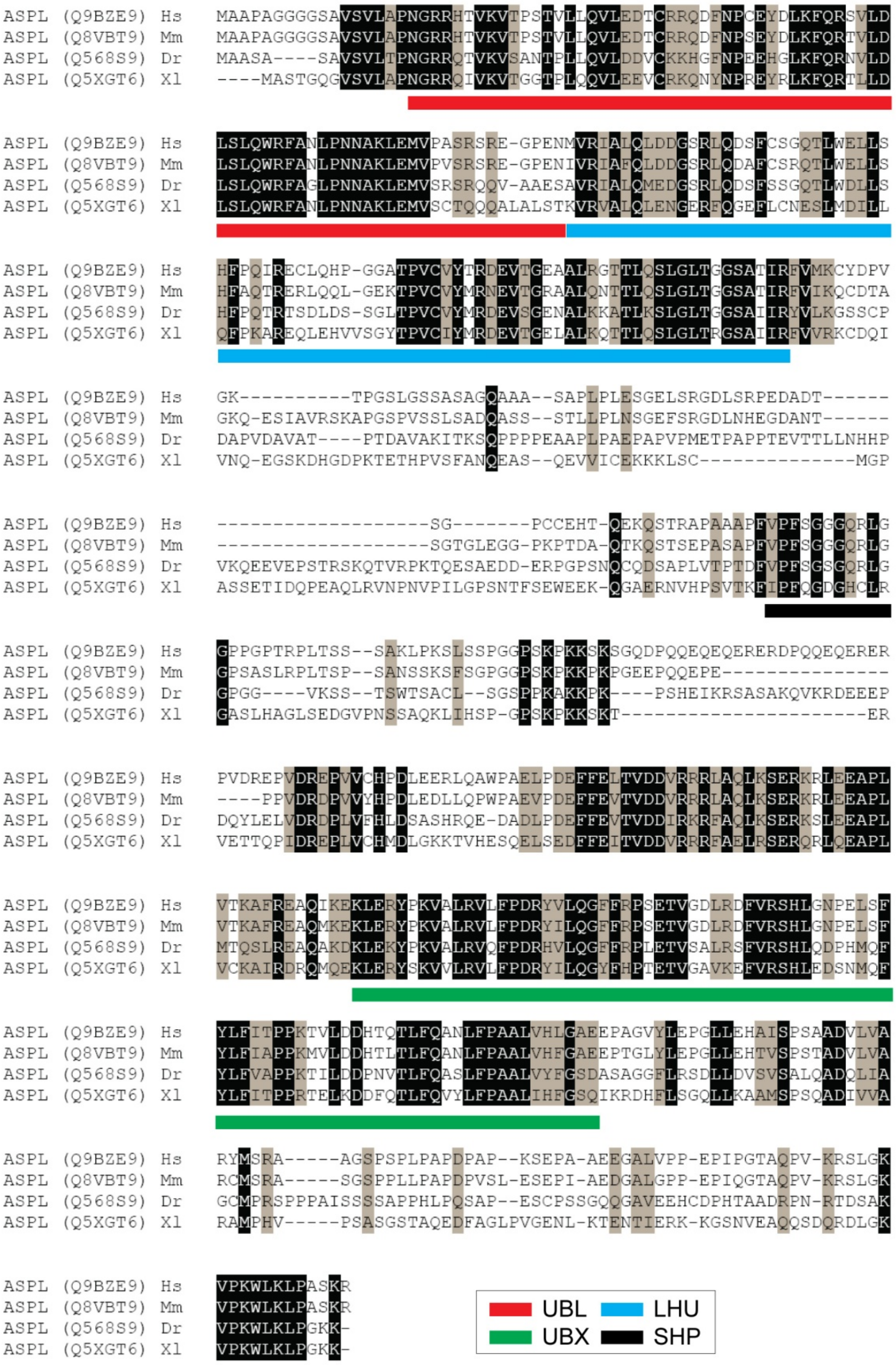

Figure S1 *ASPL is a phylogenetically conserved protein*
Clustal W (v1.82) alignment of human (Hs) ASPL with its mouse (Mm), zebra fish (Dr) and frog (Xl) orthologues. Accession numbers are given to the left. Identical and similar residues have been marked. The domain organization is indicated by the colored bars.

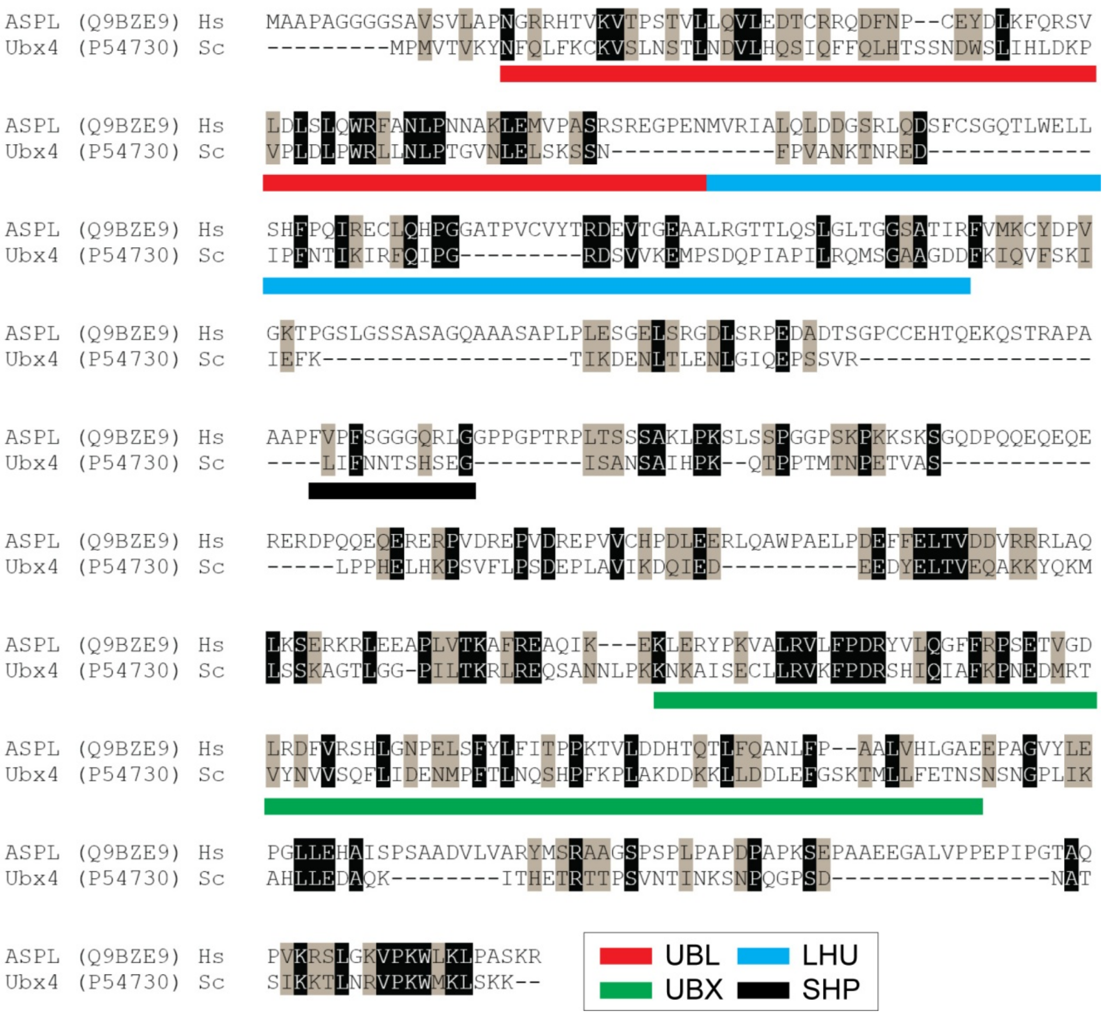

Figure S2 *Budding yeast Ubx4 is a putative orthologue of ASPL*
Clustal W (v1.82) alignment of human (Hs) ASPL with its putative budding yeast (Sc) orthologue (Ubx4). The accession numbers are given to the right. Identical and similar residues have been marked. The domain organization is indicated by the colored bars.

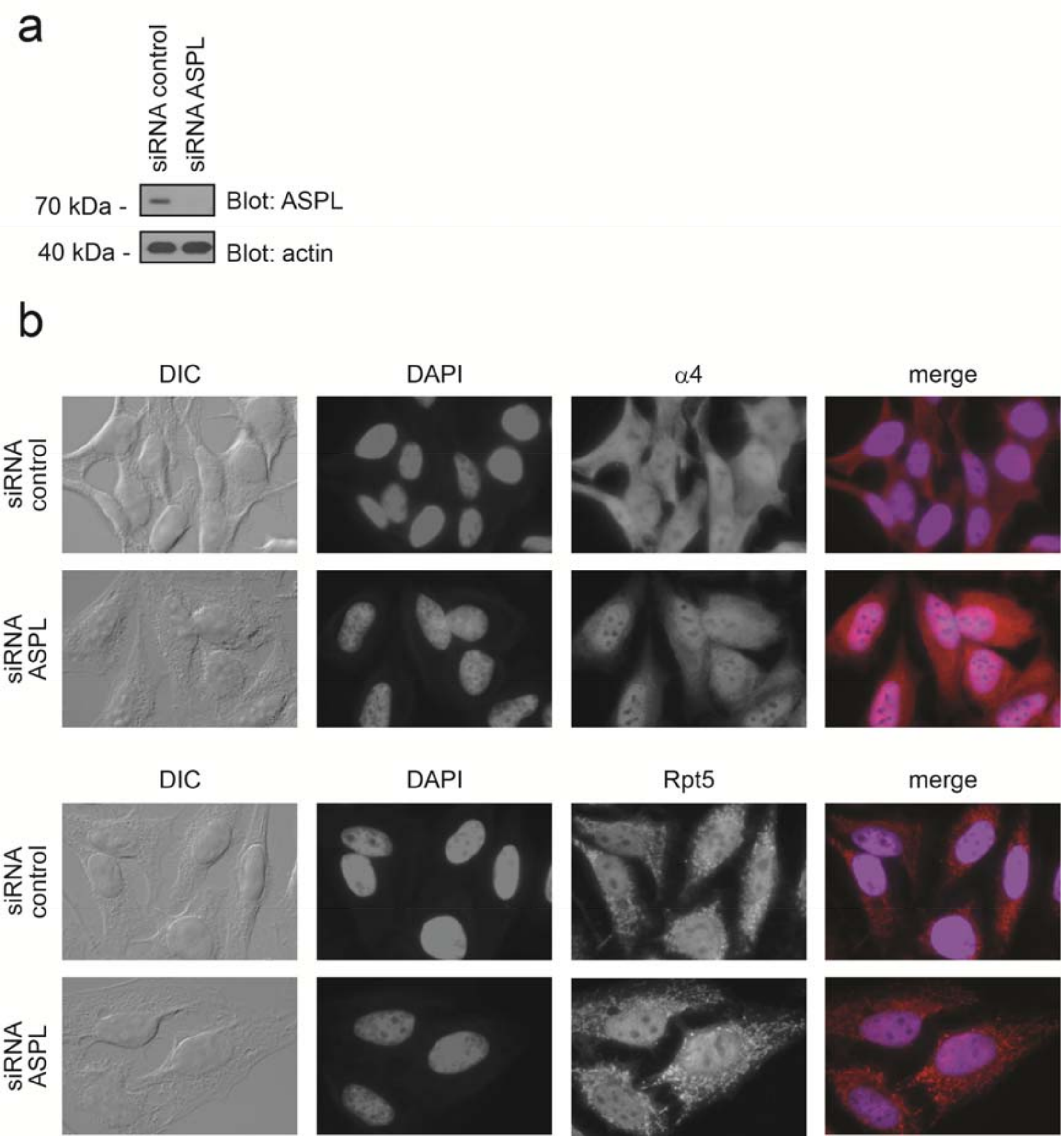

Figure S3 *Knock-down of ASPL does not affect proteasome localization in HeLa cells* (a) HeLa cells transfected with control siRNA or siRNA to ASPL were analyzed by SDS-PAGE and Western blotting using antibodies to ASPL. ASPL expression was clearly reduced in cells transfected with siRNA to ASPL. Actin served as a loading control. (b) HeLa cells transfected with control siRNA or siRNA to ASPL were analyzed by immunofluorescence microscopy using antibodies to the 20S proteasome subunit α4 (upper panels) or the 19S regulatory subunit Rpt5 (lower panels). DAPI staining was included to mark the nucleus. Both α4 and Rpt5 appear spread throughout the cytosol and nucleus, but Rpt5 seems enriched in a punctate pattern outside the nucleus. No difference in localization was observed upon ASPL knock-down.
